# Draft Genome Sequence of the Shellfish Larval Probiotic *Bacillus pumilus* RI06-95

**DOI:** 10.1128/genomeA.00858-15

**Published:** 2015-09-03

**Authors:** Meagan Hamblin, Edward Spinard, Marta Gomez-Chiarri, David R. Nelson, David C. Rowley

**Affiliations:** a Department of Biomedical and Pharmaceutical Sciences, University of Rhode Island, Kingston, Rhode Island, USA; b Department of Cell and Molecular Biology, University of Rhode Island, Kingston, Rhode Island, USA; c Department of Fisheries, Animal and Veterinary Sciences, University of Rhode Island, Kingston, Rhode Island, USA

## Abstract

*Bacillus pumilus* RI06-95 is a marine bacterium isolated in Narragansett, Rhode Island, which has shown probiotic activity against marine pathogens in larval shellfish. We report the genome of *B. pumilus* RI06-95, which provides insight into the microbe’s probiotic ability and may be used in future studies of the probiotic mechanism.

## GENOME ANNOUNCEMENT

There is significant and growing interest in the development of aquaculture probiotics, particularly for disease management in larval production. *Bacillus pumilus* RI06-95 was isolated from the Pettaquamscutt River in Narragansett, RI, and has shown significant potential as a probiotic candidate for the shellfish aquaculture industry (1). This bacterium exhibits potent *in vitro* growth inhibition against the aquatic pathogens *Vibrio harveyi* BB120 and *Roseovarius crassostreae* CV919-312, and larvae of the eastern Oyster (*Crassostrea virginica*) pretreated with RI06-95 demonstrate greater survival than untreated larvae when exposed to the pathogen *Vibrio tubiashii* RE22 (1). Here we announce the genome sequence of *Bacillus pumilus* RI06-95 to encourage investigation into the biosynthetic pathways and potential probiotic mechanism of this organism.

Three colonies of RI06-95 were grown overnight in yeast peptone broth plus 3% NaCl (YP3) (1) at 25°C with shaking. DNA was isolated using the Wizard genomic DNA purification kit (Promega) following the manufacturer’s instructions, except DNA was eluted using 100 µL of type I water. Sequencing was performed using an Illumina MiSeq sequencer at the Rhode Island Genomics and Sequencing Center. The read library contained 8,784,938 paired-end and mate-paired reads that averaged 238.79 bp in length. Reads were trimmed for quality, ambiguous nucleotides, adapter sequences, and length using CLC Genomics Workbench v. 8.0.1 (CLC Bio/Qiagen). *De novo* assembly was performed and resulted in 16 contigs with an average coverage of 913×. The total size of the draft genome is 3,643,624 bp with an average contig length of 227,727 bp and a G+C composition of 41.61%. All contigs were submitted to RAST (Rapid Annotation using Subsystem Technology) (2), which identified 3,754 open reading frames and 454 subsystems. The closest neighbor identified by SEED viewer 2.0 (3) was *Bacillus pumilus* SAFR-032 (score = 517).

The genome of *B. pumilus* RI06-95 includes a siderophore assembly subsystem, indicating the ability to compete with other organisms to sequester iron in the environment. RI06-95 also possesses genes indicative of a sialic acid metabolism, transport, and synthesis, including *NeuC* and *NeuB*. Sialic acid may be used to avoid inducing an innate immune response in a host and play a role in colonization (4). Several putative chemotaxis regulators were identified, one of which was an aerotaxis chemoreceptor protein, which orients taxis to oxygen-rich areas of media or seawater (5). Additionally, an exopolysaccharide biosynthesis cluster suggests biofilm-forming ability (6). Genes indicative of beta-lactamase production, bacitracin stress response, and fluoroquinolone resistance were also revealed.

After RAST annotation, the contigs were submitted to antiSMASH 3.0.1 (Antibiotics and Secondary Metabolite Analysis Shell) (7), which identified 10 secondary metabolite gene clusters. A cluster of particular interest, 85,837 to 144,506 nt on contig 8, encodes a nonribosomal peptide synthetase (NRPS) and type 1 polyketide synthase (t1PKS) and shows structural similarity to the putative amicoumacin gene cluster (8). Amicoumacin is an isocoumarin compound conserved in *Bacillus* sp. that has demonstrated antibacterial activity (9).

### Nucleotide sequence accession numbers.

This whole-genome shotgun project has been deposited in DDBJ/ENA/GenBank under the accession no. LFGZ00000000. The version described in this paper is the first version, LFGZ01000000.
